# Further Tests of the Utility of Integrated Speed-Accuracy Measures in Task Switching

**DOI:** 10.5334/joc.6

**Published:** 2018-01-12

**Authors:** André Vandierendonck

**Affiliations:** 1 Ghent University, BE

**Keywords:** integrated speed-accuracy scoring, task switching

## Abstract

Speed and accuracy of performance are central to many theoretical accounts of cognitive processing. In recent years, several integrated performance measures have been proposed. A comparative study of the available measures [Vandierendonck, A. (2017). A comparison of methods to combine speed and accuracy measures of performance: A rejoinder on the binning procedure. *Behavior Research Methods, 49*, 653–673. DOI: https://doi.org/10.3758/s13428-016-0721-5] concluded that three of the measures, namely inverse efficiency score, rate correct score, and linear integrated speed-accuracy score achieved a balanced integration of speed and accuracy. As a follow-up on that study, these three measures were examined in data analyses from 13 (published and unpublished) experiments in the context of task switching. The correlations of the effect sizes in these integrated scores with the effect sizes obtained in latency and accuracy were high, but varied across the three integrated measures. The efficiency to detect effects supported by the speed and accuracy data was examined by means of signal detection analyses. The three measures efficiently detected effects present in either speed or accuracy, but the rate correct score was less efficient than the other two measures and it signalled a larger number of strong effects unsupported by the speed and accuracy data. It is concluded that while the rate correct score is better avoided, and the usage of the inverse efficiency score should be restricted to data with low overall error rates, the linear integrated speed-accuracy score proves to be valid.

## Introduction

Performance on cognitive tasks can be expressed in terms of speed and accuracy of execution, typically measured by reaction time (RT) and proportion of errors (PE). Several measures that combine speed and accuracy into a single measure have been proposed (e.g., Hughes, Linck, Bowles, Koeth, & Bunting, 2014; Thorne, 2006; Townsend & Ashby, 1978; Woltz & Was, 2006). Nevertheless, researchers have been reluctant to use these measures (but see Bruyer & Brysbaert, 2011; Draheim, Hicks, & Engle, 2016), possibly because of a lack of knowledge about the usefulness of such combined measures. Following up on a recent study that showed that at least some of these measures are useful under appropriate conditions of use (Vandierendonck, 2017), the present article further examines the utility and the limits of some integrated measures.

The oldest of the integrated measures and the only one available until the beginning of the twenty-first century is the *inverse efficiency score* (IES, Townsend & Ashby, 1978). This measure takes the ratio of the average correct RT (RT*_c_*) and the proportion of correct responses:

1 {\rm{IES}} = \frac{{{\rm{RT}}_c}}{{1 - {\rm{PE}}}}

This measure is an estimation of RT adapted for the frequency of incorrect responses. For example, an average correct RT of 600 ms obtained while committing a proportion of 0.10 errors, produces an IES of 667 (ms). Experience with the usage of this measure has provided mixed results (e.g., Bruyer & Brysbaert, 2011). This measure seems to be arbitrary in the sense that there is no way of knowing whether the two performance aspects have equal impact on the end result.

Three decades after the introduction of IES, Woltz and Was (2006) proposed the rate correct score (RCS) which can be defined as:

2 {\rm{RCS}} = \frac{{\left({1 - {\rm{PE}}} \right)}}{{{\rm{RT}}_a}}

where (RT*_a_*) is the average of all responses. This score expresses the number of correct responses produced per time unit of response activity. For example, with RT expressed as seconds, RCS is the number of correct responses produced per second of activity. Around the same time, other authors have proposed measures that are similar (e.g., Santhi & Reeves, 2004) or even identical (e.g., the throughput measure, Thorne, 2006). If the same estimate of speed were involved (or if RT*_c_* = RT*_a_*) in formulas (1) and (2), RCS would be the inverse of IES (i.e., RCS = 1/IES).

Still more recently, Hughes, Link, Bowles, Koeth and Bunting (2014) proposed a score which was intended to express the combined speed and accuracy difference between two conditions (e.g., task repetition v. task switch). To obtain this score, all correct RT differences between trials of the switch condition and the average RT of the repetition condition of all subjects are grouped into 10 deciles (bins) numbered 1 to 10 (from smallest to largest difference). For each subject, a bin score is obtained as follows:

3 {\rm{bin}}= \sum\limits_{i}^{10}i \times n_{i}+ 20 \times n_{e}

where *i* is the number of the decile, *n_i_* is the number of the subject’s RT differences falling in the decile, and *n_e_* is the number of incorrect RTs; as shown in equation (3), the latter number is multiplied by 20 (a penalty). Thus, a weighted difference score between the two conditions is obtained. As a drawback, this score neither reflects any contribution of variance in the RTs of the repetition condition nor of the errors committed in the repetition condition, but fortunately, other variants of the bin score that overcome these disadvantages are possible (Vandierendonck, 2017).

A further integrated measure was proposed based on the consideration that there is a need for a combined measure in which the two performance aspects are weighted so as to contribute equally to the composite score (Vandierendonck, 2017). This linear integrated speed-accuracy score (LISAS) is defined as:

4 {\rm{LISAS}} = {{\rm{RT}}_c}\ + {\rm{PE}} \times \frac{S_{{\rm{RT}}}} {S_{{\rm{PE}}}}

Where RT*_c_* and PE are the same as in equation (1), *S*_RT_ refers to the standard deviation of the correct RTs, and *S*_PE_ to the standard deviation of PE; all these values are calculated per condition.^1^ If *S*_PE_ and/or PE are 0, LISAS is simply equal to RT*_c_*. In equation (4), PE is weighted by the ratio of the two standard deviations. This ensures that a difference of 1 standard deviation in the RT scores is given an equal weight as a difference of 1 standard deviation in the PE scores, so that when RT and PE effects are opposite, they balance each other out. Hence LISAS is by definition a balanced combination of speed and accuracy. Like IES, it can be interpreted as RT adapted for the amount of incorrect responses.

Whether or not such integrated speed-accuracy measures are useful depends on theoretical and methodological considerations. Given the assumptions about the relationship between speed and accuracy of performance, a number of cases can be distinguished. A first possibility is that RT and PE are assumed to have different origins, prohibiting a meaningful combination of both. In categorisation tasks, for instance, as trials progress, RT becomes faster, reflecting confidence in acquired knowledge, whereas errors result from not knowing the correct categorisation rule (e.g., Falmagne, 1972; Trabasso & Bower, 1966; White, 1972). As a second possibility, it may be the case that current theory formulates predictions only for speed (e.g., lexical decision, Wagenmakers, Ratcliff, Gomez, & McKoon, 2008), or only for accuracy (e.g., reasoning, Johnson-Laird, 1999). In this case, the complementary measure has no theoretical relevance. A third and more interesting possibility is that both performance aspects are theoretically relevant. However, if the factor that affects performance produces effects on speed and accuracy that are opposite or are expected to be opposite, the usage of an integrated measure does not seem appropriate.^2^ If the effects on RT and PE are about equally strong, a theoretical effort might be needed to disambiguate the expected effects. Only if the effects on RT and PE are in the same direction, an integrated speed-accuracy measure can be useful. This still leaves a range of possible situations varying from a larger effect on RT than PE, over a nearly equal effect on RT and PE (either strong or weak) to an effect on PE but no important effect on RT. In all these situations, a combination of the two measures is meaningful and may help to decide whether the factor under investigation affects cognitive performance.

Obviously, the question about the utility of integrated measures of RT and PE is confined to the latter case where RT and PE effects occur in the same direction. However, also methodological considerations play a role in deciding whether or not combined speed-accuracy measures are useful. Acceptance of an integrated measure depends on how well it is able to detect at least the same effects that are detected by speed and accuracy measures and on the extent to which it is more statistically powerful than the composing measures. Furthermore, integrated scores that assign a much larger weight to one component (e.g., accuracy) at the expense of the other component are very efficient in the detecting effects that rely on the strongly weighted component, but are deficient in their ability to detect effects that rely more on the other component. For that reason, useful integrated measures will combine the two aspects in a fair or balanced way. In this vein, a first and important consideration concerns the measurement scales of RT and PE. Whereas RT is typically based on a fine-grained millisecond-scale, PE is usually based on a coarse scale, not only when all-or-none (correct or incorrect) scoring is used, but also when error is expressed as a degree of error. Because of this difference between the RT and PE scales, the statistical power of the RT measurements outweighs that of the PE scores, so that in practice RT usually yields more reliable and more dependable observations than PE. On this basis, some researchers may decide to rely only or most heavily on RT. Note though, that if the theory’s prediction concerns both measurement aspects, the latter choice may be disputable.

Second, the balance between speed and accuracy depends on cognitive control. As a consequence, a person can modify the importance assigned to each performance aspect at any time during a series of actions (e.g., Gratton, Coles, & Donchin, 1992). This implies that the person can (a) comply with instructions to increase speed or to increase accuracy, and (b) that there is a speed-accuracy trade-off that can be changed at any time. When requested to increase the speed of responding, subjects will usually respond faster, but as the demand to increase speed becomes stronger, the tendency to produce more errors will increase as well. In other words, while RT becomes smaller, PE grows larger. When the instructions drive responding towards extreme speed, at some point performance will simply break down (known as choking under pressure, Beilock & Carr, 2001). Conversely, when asked to be careful to produce as few incorrect responses as possible, RT will typically increase. The phenomenon of finding a balance between RT and PE, is known as speed-accuracy trade-off (SAT) and precludes a straightforward and trustworthy interpretation of observations of speed or accuracy collected under trade-off conditions. A variation of SAT was included in a Monte Carlo study of the efficiency of integrated measures and showed that when the trade-off was unrelated to the experimental factor, estimates of its effect on speed, accuracy and the integrated measures were not distorted by the trade-off (Vandierendonck, 2017, Study 3). The question remains what happens if the trade-off varies over experimental and control conditions. In short, the presence of SAT remains an important concern and little is known as to whether integrated speed-accuracy measures are able to extract relevant information that is not biased by the trade-off.

Third, irrespective of SAT, effects of instructions or task variations may result in different effects in the two measures: they may both increase, they may both decrease, or one measure may grow while the other shrinks.

A series of Monte Carlo simulations that tested the properties and the utility of IES, RCS, LISAS and 4 variations of the bin measure showed that the utility of the bin measures is rather limited due to the arbitrary penalisation of PE (Vandierendonck, 2017). The study varied effect sizes of variables, the direction of effect on RT and PE (same direction, opposing directions, effect on speed but not accuracy, and effect on accuracy bot not speed), and the speed-accuracy trade-off conditions (trade speed for accuracy, neutral, or trade accuracy for speed). Five measures (two binning variations and IES, RCS and LISAS) were efficient at detecting manipulated effects on speed and/or accuracy. However, the binning measures failed to achieve efficiency for all effects in the design, and failed to combine speed and accuracy in a balanced way. Two of the three remaining measures yielded a more or less symmetric distribution at the sample level (RCS did not), and also two of the three measures accounted for a larger part of the variance than either RT or PE (IES did not). Because of the limitations of the binning measure, the present study will focus only on the latter three measures, namely IES, RCS, and LISAS. Interestingly, in the conditions where the RT and PE effects were in the same direction, the latter three measures attained on average larger effect sizes than the maximum effect size observed in RT and PE and they did so in 15% (IES), 48% (RCS) and 62% (LISAS) of the samples.

Monte Carlo simulation provides an excellent method to examine the question whether integrated speed-accuracy measures are useful. However, simulations typically require numerous assumptions that might not be representative of the cognitive processes being modelled. Therefore, as a follow-up on the earlier simulation studies (Vandierendonck, 2017), it seems valuable to show that the integrated measures perform well in real data. Such an action could be suitably performed, as data sets based on a variation of factorial combinations of task switching with other factors are available. Hence, the main purpose of the present paper is to further examine the utility of integrated speed-accuracy measures in the domain of task switching using existing data sets.

The integrated measures were studied in two sets of data that were collected in the context of research projects on task switching in the author’s lab. In task switching research, RT and PE differences are expected between task repetition and task switching conditions and these differences are expected to be in the same direction, at least when there are no strong speed-accuracy trade-offs. In the remainder of the paper, these data sets, which include as well published as unpublished data, will be used to assess the utility of the three integrated measures that were found to be acceptable in the comparative Monte Carlo simulations of Vandierendonck (2017), namely LISAS, IES and RCS. These data sets are particularly useful for this purpose, firstly because the two data sets each consist of a series of homogenous experiments based on multi-factorial designs so that most of the experiments yield a rather large number of effects. Secondly, the effect sizes of speed and accuracy are generally pointing in the same direction (correlation of the effect sizes over the entire set amounts to 0.66) so that there is no reason to assume that these data are affected by speed-accuracy trade-offs. Nevertheless, the data sets contain a sufficient number of cases in which the RT and PE effect sizes are different. Thirdly, the effect sizes in both RT and PE span a broad range from 0 to 0.95 in RT and from 0 to 0.82 in PE. For all these reasons, tests of the utility of integrated speed-accuracy measures on these data sets seems to provide a useful complement to simulation studies.

In line with the approach taken in that study, the focus is on the efficiency with which integrated measures are able to detect effects by picking up information from the two components, speed and accuracy. The rationale is the following. The integrated measures combine RT and PE scores into a single measure, so that the combined score reflects information present in both components. These integrated measures can replace the two component measures if he information present in these components is represented equally well or even better than when only RT and PE would be used. In other words, if RT and PE reveal effects in the same direction, it is expected that the integrated measures show the combined effect, and the larger the observed effect on RT and PE, the larger the observed effect on the integrated measures is expected to be. Conversely, if the observed effect on RT and PE tends to be small, the effect on the integrated measure is also expected to be rather small. It is important to note that these relationships are situated at the level of the data sample.

## Data Sets

### Data Set 1

**Figure 1:**
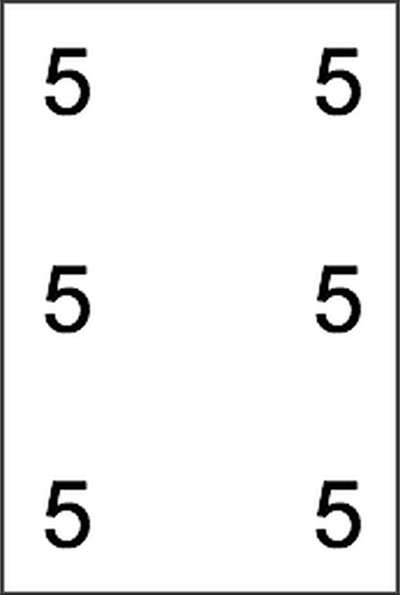
Example of a stimulus consisting of digits (2, 3, 5, or 6) in an arrangement where 2, 3, 5, or 6 digits are shown in a playing card pattern.

Switching from one task to another involves a performance cost that has been attributed to the requirement to configure another task set and shield it from interference due to overlap with the previously relevant task set on switch trials as compared to trials where the same task is repeated (for reviews, see Kiesel et al., 2010; Monsell, 2003; Vandierendonck, Liefooghe, & Verbruggen, 2010). These reconfiguration processes on switch trials require additional time (hence an RT cost) and occasionally result in errors (hence a PE cost). An important question concerns the properties of the task set, which is often considered to involve several processes or settings (Logan & Gordon, 2001; Rogers & Monsell, 1995). These settings could be organised in a hierarchical structure (e.g., Kleinsorge & Heuer, 1999; Kleinsorge, Heuer, & Schmidtke, 2001, 2002, 2004), a componential structure (e.g., Hübner, Futterer, & Steinhauser, 2001), or could form a loose structure (e.g., Allport, Styles, & Hsieh, 1994). In this context, data were collected to investigate the role of the task rule and the relevant stimulus dimension as two components of the task set that may occur in a hierarchical, componential or loose structure. Using stimuli that allow application of the same two tasks (magnitude and parity judgment) to two stimulus features (the digit or the number of digits displayed; see Figure 1 for an example), the data were collected to further test the different views on task-set organisation. Experiments 1–4 of this set were reported by Vandierendonck, Christiaens and Liefooghe (2008).

**Method.** Data set 1 includes data of 8 experiments that used the same basic building block consisting of the within-subject factors of task transition (task repetition v. task switch) and dimension transition (dimension repetition v. dimension switch). Over the experiments, several additional factors were varied, namely the cue-stimulus interval (CSI: the interval between the start of the presentation of the cue and the start of the presentation of the task stimulus), the stimuli, the cue-cue interval (CCI: the interval between the start of the first and the second cue component), the order of the cue components and the spatial organisation of the two cue components.

Figure 1 displays an example of a stimulus as used in seven of these experiments; the other experiment used global-local stimuli (cf. Navon, 1977). The stimulus in this figure is based on a playing card configuration in which the same digit is displayed a number of times. The subject’s task was to classify the stimulus either on the basis of the number of digits or on the value of the digits displayed. Classification either required a magnitude judgment (smaller or larger than 4) or a parity judgment (odd or even). On each trial, the relevant task and dimension were cued by displaying both components either in a dimension-central format (e.g., LO N HI; this particular cue signals that the number of elements in the display [N] has to be classified as small [low, LO] or large [high, HI]), or in a dimension-left format (e.g., D OD EV; D signals digit as relevant dimension and parity as the required task with categories odd [OD] and even [EV]). For more details about the procedure, the reader is referred to Vandierendonck et al. (2008).

All participants in the experiments gave written consent, and were either first-year psychology students who participated for course requirements and credit or paid volunteers recruited from the subject panel of the Department of Experimental Psychology at Ghent University. Table 1 displays more detailed information about the participants, the design and the specificity of each experiment.

**Table 1:** Specifications of the experiments in Data Set 1: number of participants, design, and specific features of the experiment.

Experiment	N	Design	Features

1	20 (14)	T × D	CSI = 0; CCI = 0
2	20 (17)	S × T × D	CSI = 300 or 1000 ms; CCI = 0
3	22 (18)	S × T × D	Same as Exp. 2; global-local stimuli
4	22 (19)	S × T × D	Same as Exp. 2; both tasks with same hand
5	48 (36)	C × S × T × D	Cue: Dimension-Left; Dimension first
6	45 (41)	C × S × T × D	Cue: Dimension-Left; Task first
7	48 (43)	C × S × T × D	Cue: Dimension-Centre; Dimension first
8	47 (36)	C × S × T × D	Cue: Dimension-Centre; Task first

*Note:* In column “N” the number between parentheses refers to the number of females in the experiment. The letters in the design specification (column “Design”) are as follows: T = Task transition; D = Dimension transition; S = CSI (300 or 1000 ms); C = CCI (0, 300 or 1000 ms).

### Data Set 2

The second data set includes experiments that were performed in the context of a research project regarding the differences between explicit cues (signalling which task has to be performed) and transition cues (signalling whether the task remains the same or changes) in task switching. Five experiments were conducted, three of which were published (Van Loy, Liefooghe, & Vandierendonck, 2010). These experiments were designed to distinguish the roles of cue-related and task-related processes in task switching and to study the basis for the occurrence of cue-task transition (in)congruency. With explicit cues, the cue specifies the task so that when the cue changes, the task also changes. Transition cues, in contrast, specify the task transition: same or different. As a consequence, the cue transition (whether the cue is the same as before) can be congruent or incongruent with the task transition (repeat or switch): in congruent transitions the cue and the task either both change or both remain the same, when only one of both transitions is a change, the cue and task transitions are incongruent.

**Method.** In each experiment, subjects were presented digits 1–9, excluding 5 and performed either a magnitude decision (smaller or larger than 5) or a parity decision. At the start of every trial, a cue indicated which of these tasks was to be performed. Experiments 1–3 orthogonally crossed the factors Cue type (explicit v. transition cue) and Transition type. Four types of transitions were defined on the basis of the sequence of events leading up to the present trial. Labelling the two tasks A and B, these four transition types are: AAA, BAA, ABA, and BBA. When transition cues are used (SAME or DIFFERENT), these four transitions involve respectively, complete repetition (cue and task repeat), cue switch (task repeats, but cue changes), task switch (the cue repeats, but the task changes) and complete switch (both the cue and the task change). Experiments 4 and 5 also included the factor transition type crossed with another factor (see below).

Whereas Experiment 1 was designed to simply compare the two types of cueing, it involved one further within-subject factor, namely response congruency. When the two tasks require the same response to the present stimulus, the response is congruent. For example, with the response mappings small-left (large-right) and odd-left (even-right), the digit 3 requires a left response for both tasks (congruent), whereas the digit 4 requires different response for the magnitude than for the parity task. Experiments 2–5 used the so-called double registration procedure (Arrington, Logan, & Schneider, 2007) which requires two responses in every trial, namely a response to the cue and a response to the target stimulus. The response to the cue requires the subject to indicate which of the tasks must be performed in the trial, thus showing understanding of the meaning of the cue. As a consequence, for each of these experiments two dependent variables were analysed. In Experiments 4 and 5, the task indication response was either a choice response (indicating the task to be performed) or a simple response (indicating that the cue was understood). In Experiment 4 the indication response was manual, in Experiment 5 it was a vocal response.

All participants in the experiments were first-year psychology students who participated for course requirements and credit. More details about the experiments are shown in Table 2. For a full explanation, the reader is referred to Van Loy et al. (2010).

**Table 2:** Specifications of the experiments in Data Set 2: number of participants, design, and specific features of the experiment.

Experiment	N	Design	Features

1	24 (15)	C × T × I	I refers to response (in)congruency (do tasks require same response or not)
2	24 (20)	C × T	500 ms delay between indication response and task stimulus
3	20 (19)	C × T	Same as Exp. 2 with no delay
4	19 (16)	R × T × I	Indication: choice or simple manual response
5	18 (17)	R × T × I	Indication: choice or simple vocal response

*Note:* In column “N” the number between parentheses refers to the number of females in the experiment. The letters in the design specification (column “Design”) are as follows: C = Cue type (explicit or transition), T = transition type (repetition v. switch); R = response type (choice or simple), I = response congruency across tasks.

### Data analysis

In all experiments of both data sets data analyses were performed on each of five measures, namely RT*_c_*, PE, LISAS, IES and RCS. For the analysis of RT*_c_*, incorrect trials and trials following an incorrect trial were excluded (a standard procedure in the analysis of task switching data). IES was calculated per cell of the design for each subject on the basis of the means of the correct RT and PE data in that cell. The same procedure was followed for LISAS with the provision that also the standard deviations of RT and PE were calculated per subject per cell. For RCS, the procedure was the same as for IES but with the average of all RTs instead of only correct RTs.

In Data Set 1, two analyses were performed on each measure: an ANOVA involving all the factors of the design and their interactions as shown in Table 1, and a test of a series of planned contrasts^3^ on the means of the Task × Dimension building block and the interactions of these contrasts with the other factors in the design.

In Data Set 2, an ANOVA tested the main effects and interactions of the design as specified in Table 2 with only two levels for the factor Transition type (complete repetition or complete switch). In addition, a contrast analysis was performed on the subset of the data with transition cues. For the contrast analysis, three orthogonal contrasts were used, namely cue repetition v. cue switch, task repetition v. task switch and the interaction of these two. The interactions of the contrasts with other factors in the design were also included (in Experiments 1, 4 and 5).

Because all the designs in the present paper involve within-subject factors, all analyses were performed by means of the multivariate general linear model with contrasts in the dependent variables to avoid problems with the sphericity assumption of the ANOVA model. For all the effects tested in all these analyses, \eta _p^2 was used as an estimate of the effect size. These effect sizes constitute the basic data for the tests on the validity of integrated speed-accuracy measures.

## Results and Discussion

### Detection efficiency

**Figure 2:**
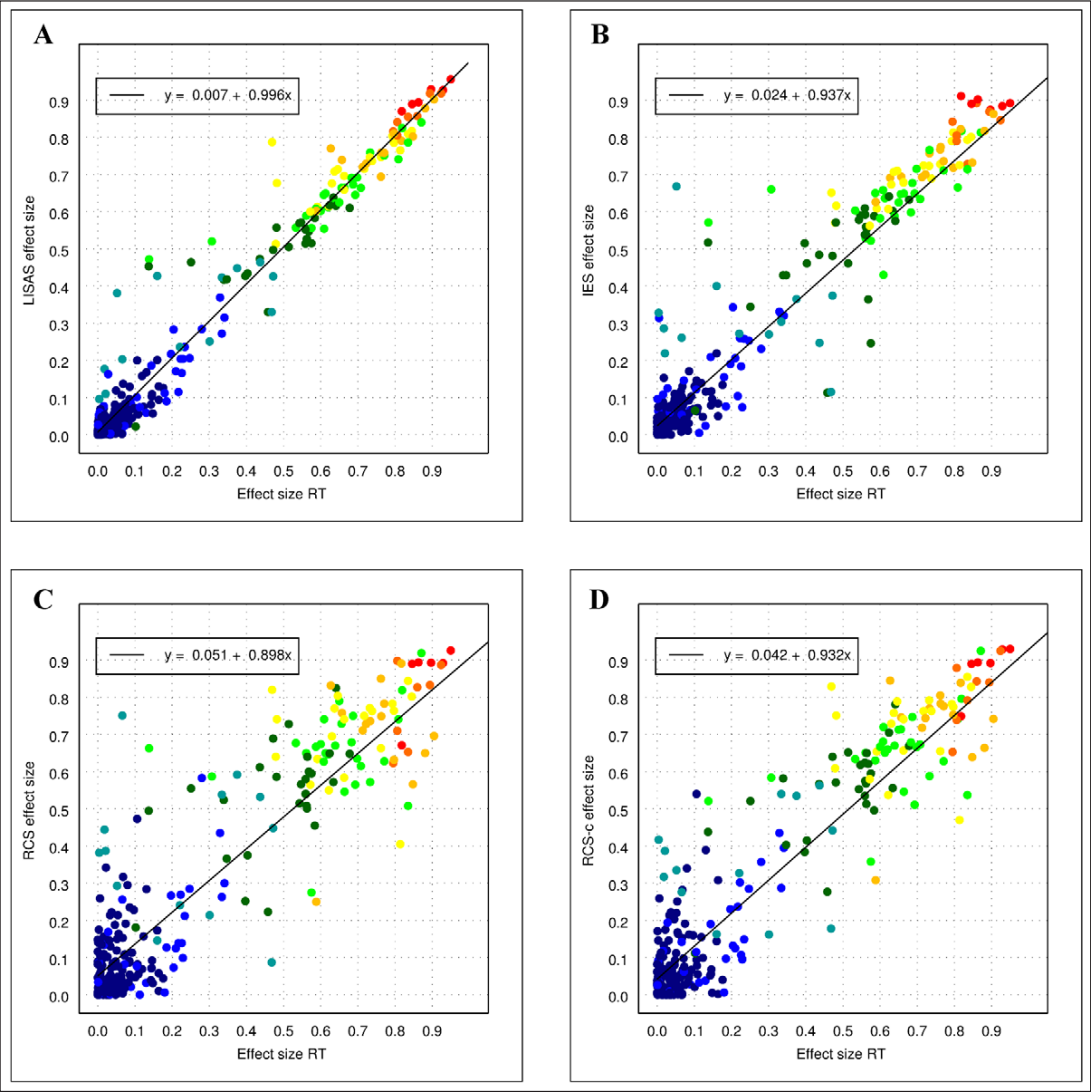
Effect sizes of all the effects in both Data Sets in LISAS **(panel A)**, IES **(panel B)**, RCS **(panel C)**, and RCS-c **(panel D)** as a function of the effect size in RT. The colours of the plotted points indicate the average effect size obtained by RT and PE: dark blue (up to 0.1), blue (<0.2), cyan (<0.3), dark green (<0.4), green (<0.5), yellow (<0.6), orange (<0.7), red orange (<0.8) and red (<1.0).

**Figure 3:**
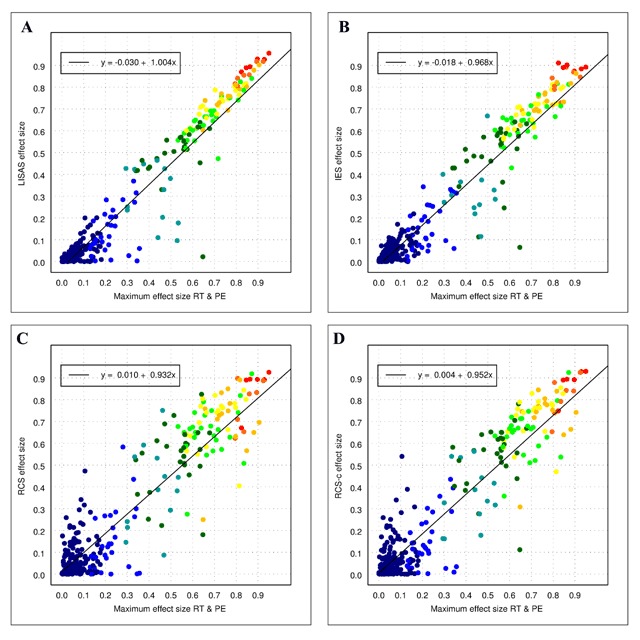
Effect sizes of all the effects in both Data Sets in LISAS **(panel A)**, IES **(panel B)**, RCS **(panel C)**, and RCS-c **(panel D)** as a function of the maximal effect size in RT and PE. The colours of the plotted points indicate the average effect size obtained by RT and PE: dark blue (up to 0.1), blue (<0.2), cyan (<0.3), dark green (<0.4), green (<0.5), yellow (<0.6), orange (<0.7), red orange (<0.8) and red (<1.0).

The utility of the integrated measures was assessed by comparing the results of the data analyses on these measures with the outcomes of the composing measures. A first analysis examined to which degree effects in these measures are recovered by each of the three integrated measures. Considering that RT is a statistically more sensitive measure than PE and has typically larger effect sizes than PE, plotting the effect sizes of the integrated speed-accuracy (ISA) measures against the RT effect sizes, allows for an examination of the consistency between the measures. Figure 2 displays the effect size of the integrated measures as a function of the effect size obtained in RT. In order to counter the presumption that PE effects play no role, Figure 3 shows the effects sizes as a function of the maximal effect size obtained in RT and PE. In these displays, each point in the scatter plots represents an effect in the analyses of the experiments in Data Sets 1 and 2 (296 effects in all). As can be seen in these figures, the effect sizes in the integrated measures tended to vary linearly with the effect sizes in RT as well as with the maximum of the effect sizes in RT and PE. Table 3 shows the corresponding correlations for Figure 2 and their 0.95 confidence intervals. The table indicates that the correlation between RT and LISAS was significantly higher than that of the two other ISA measures, and that the correlation between RT and IES was larger than that between RT and RCS. These significant differences were confirmed also in the partial correlations with PE held constant. The correlations between the effect sizes of PE and the integrated measures were not significantly different from each other, whereas the partial correlations with RT held constant showed that the partial correlation of PE with RCS was significantly lower than the partial correlation of PE with the other two measures.

**Table 3:** Product-moment correlations and partial correlations (holding either PE or RT constant) of the integrated measure with RT and PE and their 0.95 confidence intervals.

	Correlations						Partial Correlations					

	RT			PE			RT-PE			PE-RT		

ISA	Value	CI		Value	CI		Value	CI		Value	CI	

LISAS	0.981	0.976	0.985	0.738	0.681	0.786	0.972	0.965	0.978	0.592	0.513	0.662
IES	0.956	0.945	0.965	0.765	0.713	0.808	0.930	0.913	0.944	0.592	0.512	0.661
RCS	0.911	0.890	0.929	0.719	0.659	0.770	0.835	0.797	0.867	0.371	0.268	0.465

*Note:* The correlations are based on 296 observations. The columns under the header RT-PE show the partial correlations of RT with the integrated measures with PE held constant; likewise, the columns under the header PE-RT show the partial correlations of PE with the integrated measures with RT held constant.

Both figures further indicate that the variability of the data points around the linear regression line tended to be smaller in LISAS than in IES, and was larger in RCS than in the two other measures. In order to test the strength of these observed differences, the RT effect sizes were partitioned into deciles. The averages and the standard deviations of the corresponding ISA effect sizes were calculated per decile for each of the two data sets separately. These data were subjected to a 2 (Data Set) × 3 (Effect Level: deciles 1–3, 3–6, and 7–10) × 3 (ISA measures) orthogonal ANOVA with repeated measures on the last factor. The analysis based on the means revealed no differences except for Effect Level. The ANOVA on the standard deviations per decile, revealed a significant main effect of Data Set (M = 0.054 and 0.120 for respectively set 1 and 2), *F*(1,14) = 24.69, *p* < 0.001, \eta _p^2 = 0.64. The standard deviations also varied with Effect Level (M = 0.065, 0.101, and 0.093), *F*(2,14) = 6.82, *p* < 0.01, \eta _p^2 = 0.49. Also the main effect of ISA Measures was significant (M = 0.060, 0.085 and 0.116 for LISAS, IES and RCS, respectively), *F*(2,13) = 48.09, *p* < 0.001, \eta _p^2 = 0.88. The contrast between RCS and the other two was significant, *F*(1,14) = 15.85, *p* < 0.001, \eta _p^2 = 0.53, as was the contrast between LISAS and IES, *F*(1,14) = 21.80, *p* < 0.001, \eta _p^2 = 0.61. Measures interacted with Data Set, *F*(2,13) = 10.81, *p* < 0.01, \eta _p^2 = 0.62. To better understand this interaction, it was also decomposed by taking the interaction of Data Set with the two contrasts on Measures. The RCS-other contrast did not interact with Data Set, *F* < 1, but the contrast between LISAS and IES did, *F*(1,14) = 9.91, *p* < 0.01, \eta _p^2 = 0.41, with a very small difference in Data Set 1 (M = 0.038 for LISAS and 0.046 for IES) and a large difference in Data Set 2 (M = 0.081 and 0.125, respectively).

A rather striking feature in Figures 2 and 3 concerns the larger variability of the effect sizes corresponding to the lower values on the x-axis: at this lower end of the continuum, some of the effects of the integrated measures seem to be rather high. Although these rather large deviations from the regression line are present in the panels of the three measures, the frequency of these deviations tends to be larger in IES and RCS than in LISAS. A further examination of these observations tested how well the integrated measures were able to recover the effects present in the component measures by means of a signal detection analysis.

### Signal detection analysis

Per experiment, the entire set of effects was partitioned on the basis of the effect size of RT and PE into a category with small and one with large effects. The small category contained the effects for which the effect sizes of RT and PE were both smaller than a predefined criterion. The other effects (either RT or PE or both at least as large as the criterion) were assigned to the large category. The choice of a criterion is to some extent arbitrary, but considering that the integrated measures are based on the same information as RT and PE, it may be expected that these measures report a reliable effect when either RT or PE or both effects are reliable. For that reason, the significance threshold was used, and the criterion value was defined to correspond to the size of an effect with one degree of freedom at the significance level of *α* = .05. Thus, the large category included all the effects which were larger than or equal to this critical value in either RT or PE and may also contain some nonsignificant effects with more than one degree of freedom; the small category included all the other effects (i.e., in which \eta _p^2 was smaller than the critical value in both RT and PE).^4^ For each integrated measure, the effects in the large category were categorised as hit (effect size equal or larger than the criterion) or miss (effect size smaller than the criterion). Likewise, the effects in the small category were categorised as false alarm when the effect size was larger than or equal to the criterion and as correct rejection otherwise. The proportions of effects in each of these categories were then calculated. The proportions of hits and false alarms were used to calculate the sensitivity of detection (*d’*).

For each experiment, Table 4 shows per integrated measure the proportion of hits and false alarms and the resulting *d’* value. At the bottom of the table, the collapsed values over all experiments are shown. The program of Van der Goten and Vandierendonck (1997) was used to calculate *d’*. Because the number of effects is rather small in some of the experiments, there is a risk that some proportions that are needed for the calculation of *d’* amount to 0 or 1. Such values result in infinite *d’* scores. This can be avoided by taking 1/(2N) or 1–1/(2N) instead of 0 and 1 respectively, where N is the total number of cases (Macmillan & Kaplan, 1985) or by adding 0.5 to all four cells of the 2 × 2 matrix on which the sensitivity measure is based (Snodgrass & Corwin, 1988). The latter method was used in the present analyses because simulation work has demonstrated that the latter method, often used in log-linear analyses, yields less biased results than the first method (Hautus, 1995).

**Table 4:** Proportions of effects detected by LISAS, IES, and RCS when a critical effect was detected by either RT or PE (hits) and when neither RT and PE detected a critical effect (false alarms) in both datasets.

Exp	Effects (N)	Large	Small	LISAS			IES			RCS		

				H	FA	*d’*	H	FA	*d’*	H	FA	*d’*

Data Set 1												

1	7	4	3	0.90	0.13	2.43	0.90	0.13	2.43	0.90	0.38	1.60
2	15	6	9	0.79	0.05	2.44	0.79	0.15	1.83	0.79	0.15	1.83
3	15	5	10	0.92	0.05	3.07	0.92	0.05	3.07	0.92	0.32	1.86
4	15	7	8	0.81	0.06	2.48	0.81	0.06	2.48	0.69	0.06	2.08
5	35	15	20	0.91	0.07	2.78	0.78	0.07	2.24	0.84	0.17	1.98
6	35	14	21	0.70	0.02	2.52	0.77	0.11	1.94	0.70	0.30	1.06
7	35	15	20	0.53	0.07	1.54	0.78	0.07	2.24	0.78	0.07	2.24
8	35	11	24	0.79	0.02	2.87	0.79	0.02	2.87	0.71	0.38	0.85
**Data Set 2**												

1	16	13	3	0.82	0.13	2.07	0.96	0.13	2.95	0.89	0.63	0.92
2.1	7	6	1	0.79	0.25	1.47	0.93	0.25	2.14	0.93	0.75	0.79
2.2	7	5	2	0.75	0.17	1.64	0.75	0.17	1.64	0.75	0.17	1.64
3.1	7	6	1	0.79	0.25	1.47	0.79	0.25	1.47	0.93	0.75	0.79
3.2	7	4	3	0.70	0.13	1.67	0.90	0.13	2.43	0.90	0.38	1.60
4.1	17	11	6	0.71	0.07	2.01	0.79	0.07	2.28	0.71	0.07	2.01
4.2	17	14	3	0.97	0.13	2.98	0.90	0.13	2.43	0.90	0.13	2.43
5.1	13	9	4	0.85	0.10	2.32	0.75	0.10	1.96	0.75	0.10	1.96
5.2	13	8	5	0.83	0.08	2.35	0.83	0.08	2.35	0.83	0.08	2.35

Total	296	153	143	0.82	0.02	3.03	0.86	0.04	2.85	0.84	0.20	1.82

*Note:* Each row in the table reports the data of one of the experiments in this dataset. The column “Effects” contains the number of effects that were evaluated in the analysis of the experiments. The “Large” and “Small” columns, contain the number of effects in which either RT or PE were significant or neither was significant. The columns H and FA respectively contain the proportions of hits and false alarms. These proportions are corrected in line with the calculation procedure as explained in the text. The column *d’* reports signal detection sensitivity. In the row “Total” the values are not the averages over the rows, but the values calculated on the entire set of analyses in the 5 experiments. The rows 2.1, 3.1, 4.1 and 5.1 show the results of the analysis of the indication response, whereas row 2.2, 3.2, 4.2, and 5.2 contain the results of the analysis of task response.

A 2 (Data Set) × 3 (Measures) ANOVA with repeated measures on the last factor was performed on the *d’* values. Only the main effect of measures was significant, *F*(2,14) = 9.03, *p* < 0.01, \eta _p^2 = 0.56. The contrast between RCS and the other two measures was reliable, *F*(1,15) = 18.32, *p* < .001, \eta _p^2 = 0.55, but the contrast between LISAS and IES was not, *F* < 1. From Table 4 it is also clear that the proportion of false alarms was much larger in RCS than in LISAS and IES. Application of the *χ*^2^ statistic confirmed this difference, *χ*^2^(1) = 41.41, *p* < 0.001 (with Yates correction). The question remains whether this difference is a consequence of tendency of RCS to produce larger effects generally, or whether these large effects are indeed best considered as false alarms.

### Effect size differences in signal detection categories

If it is the case that RCS effect sizes tend to be larger than those obtained by the other measures, it is possible that the larger number of false alarms in RCS is the result of a tendency in RCS to produce higher effect sizes than the composing measures, with as a consequence that the higher effect size just exceeds the critical value for the large category. If so, the false alarms would be the result of a positive feature of RCS. Is it indeed the case that the occurrences of false alarms constitute an artefact?

In order to test this speculation, the RCS effect sizes in the hit, miss, false alarm and correct rejection categories were compared to the maximum effect size of RT and PE. Table 5 displays the mean effect sizes per category for both sets of effects (RCS and maximum of RT and PE). These data were subjected to a 4 (Category) × 2 (Measures) ANOVA with repeated measures on the last factor. The main effect of Category and its interaction with Measures was significant, but the main effect of Measures not reliable: *F*(3,292) = 296.20, *p* < 0.001, \eta _p^2 = 0.75 for Category (M = 0.58, 0.14, 0.12, and 0.05 for hit, miss, false alarm and correct rejection, respectively), *F*(1,292) = 2.66, *p* = 0.10, \eta _p^2 = 0.01 for Measures (M = 0.301 for the maximum of RT and PE and 0.290 for RCS), and *F*(3,292) = 38.43, *p* < 0.001, \eta _p^2 = 0.28 for their interaction. Within the large category (hits and misses), the two measures differed with respect to the effect size level in the miss category but not in the hit category, as can be seen in Table 5; this is confirmed by the interaction of the hit-miss contrast with Measures, *F*(1,292) = 43.37, *p* < 0.001, \eta _p^2 = 0.13. Within the small category, the difference between the measures was in opposite directions in false alarms and correct rejections (see Table 5); the interaction of Measures with the contrast between false-alarm and correct rejections was significant, *F*(1,292) = 56.16, *p* < 0.001, \eta _p^2 = 0.16. This analysis shows that on average the RCS effect sizes did not differ from those of RT and PE, while, on the one hand, the average level of the misses and the correct rejections was much lower than that in RT and PE, and on the other hand, the average effect size level of false alarms was much larger than that of RT and PE. This suggests that the high false alarm rate is RCS is not due to a general tendency to produce higher effect sizes in all categories. In fact, only in the false alarms category the average effect size was higher than that of the maximum of RT and PE. A similar analysis with LISAS and IES was not performed because of the low absolute number of false alarms in these measures (respectively 2 and 5).

**Table 5:** Mean effect size (standard errors between brackets) of RCS and the maximum of RT and PE as a function of hits, misses, false alarms and correct rejections in RCS.

	Hit	Miss	False Alarm	Correct Rejection

Max (RT, PE)	0.586 (0.019)	0.217 (0.027)	0.058 (0.007)	0.057 (0.005)
RCS	0.576 (0.020)	0.065 (0.013)	0.191 (0.017)	0.039 (0.004)

The question may be raised why the performance of RCS deviates from that of the other two measures. One factor which may contribute to these differences is the fact that RCS is based on all RTs whereas IES and LISAS are calculated from correct RTs only. In order to examine whether this factor accounts for the observed difference, a new version of RCS (RCS-c) was calculated which is based on the correct RTs completely in the same way as the other two measures. The scatterplots of the effect sizes against the effect size of RT are shown in panel D of Figure 2 and against the maximum of RT and PE in panel D of Figure 3. In comparison to RCS, RCS-c shows a smaller dispersion around the regression line. However, rather obvious deviations are present at the lowest RT effect sizes, and overall the variability still seems to be larger than for IES and LISAS. In the analysis of the standard deviations of the effect sizes, the contrast between RCS and RCS-c (Ms = 0.116 and 0.104) failed to attain significance, *F*(1,14) = 3.47, *p* = 0.08, \eta _p^2 = 0.20. The difference with IES (M = 0.085) is striking, because RCS-c is effectively 1/IES. That the variability in RCS-c also differs from that of IES is probably related to the fact that division by RT increases the skewness (cf. the remarks on skewness of RCS in Vandierendonck, 2017).

Average *d’* of RCS-c was larger (M = 1.74) than that of RCS (M = 1.55), *F*(1,15) = 14.55, *p* < .01, \eta _p^2 = 0.49, but it was still smaller than that of the other measures (M = 2.52 and 2.47 for LISAS and IES), *F*(1,15) = 10.19, *p* < .01, \eta _p^2 = 0.41. The overall proportion of false alarm dropped from 0.22 for RCS to 0.18 for RCS-c, and this drop was not significant, *χ*^2^(1) = 0.35, *p* > 0.50.

The 4 (detection Category) × 2 (Measures) ANOVA was repeated with RCS-c instead of RCS. If anything, this analysis revealed the same significant effects as the analysis on RCS, except that most of the effects were even stronger. Measures interacted with the contrast of the hit and miss categories, *F*(1,292) = 43.69, *p* < 0.001, \eta _p^2 = 0.13 (M = 0.196 and 0.061 for the maximum of RT and PE and RCS-c respectively). Measures interacted also with the contrast of false alarm and correct rejection categories, *F*(1,292) = 70.89, *p* < 0.001, \eta _p^2 = 0.20. As for RCS, the differences between the measures were in opposite directions in these two categories: respectively M = 0.061 v. 0.207 in the false alarm, and 0.057 v. 0.038 in the correct rejection category.

All these analyses confirm that the problems with RCS (and the throughput measure) are not due to the fact that all RTs instead of only correct RTs are used to calculate the score. Using a score which is effectively the inverse of IES did not remove the variability in the generated effect sizes nor the occurrence of rather deviant false alarm effects (on average 3 times the size of the maximum of RT and PE).

## General Discussion

### Summary

The findings can be summarised as follows. First, the effect sizes of the integrated speed-accuracy measures showed a strong linear relationship with RT effect sizes and a somewhat less strong relationship with PE effect sizes. Although the correlations were high for all three measures, they were different across the three integrated measures. Second, the scatter plots revealed important differences in effect size variability among the three measures with the smallest degree of variability in LISAS and the largest in RCS, with IES in between. Moreover, at the lower end of the RT effect size continuum, RCS produced an important number of rather large effects. Third, signal detection analysis confirmed that the sensitivity (*d’*) of RCS was smaller than that of LISAS and IES, which were about equally sensitive. This poorer sensitivity on the part of RCS was mainly caused by the presence of a rather large number of false alarms. Fourth, a comparison of the RCS effect sizes to the maximum of RT and PE confirmed that the RCS effect sizes were larger than those of the composing measures only in the false alarm category and about equal or even smaller in the other categories. Finally, an additional test with a variant of RCS calculated on the basis of correct RTs instead of all RTs, did not at all result in improved detection performance.

### Discussion

These findings confirm that at least some of the integrated speed-accuracy measures are useful in situations where speed and accuracy effects are more or less pointing in the same direction. Yet, not all integrated measures that have been proposed thus far appear to be recommendable. Previous work has already elucidated the drawbacks of the binning procedure for the variant proposed by Hughes et al. (2014) as well as the variants that were introduced to overcome the difficulties raised by the original measure (Vandierendonck, 2017). Thus far, also some doubts have been raised about the general usefulness of IES (Bruyer & Brysbaert, 2011; Hughes et al., 2014; Vandierendonck, 2017), while RCS was considered trustworthy (Hughes et al., 2014), even though its statistical distribution was skewed at the sample level (Vandierendonck, 2017). The present study does not support these earlier assessments of the benefits of RCS. To the contrary, the findings clearly show that (a) RCS produces quite variable results over the entire spectrum of effect sizes (cf. the scatter plots in Figure 2C, 2D and Figure 3C, 3D), and (b) RCS generates rather strong effects without any support for such a claim in RT and PE. If it were the case that small effects in RT and PE would result in a small but reliable effect in the integration of both, this measure would be praised as bringing clarity in situations where this is needed and useful. Yet, the analysis of the effect sizes in the different categories shows that the proportion and the size of the effects in the false alarm category deviates from the effect sizes in the other categories. Moreover, the experience with the variant of RCS where instead of all RTs only the correct RTs are used to calculate the integrated score shows that the problem with RCS is more general and is quite likely related to the earlier observation that the RCS scores have a strong positive skewness at the sample level, while for the other measures the initial skewness in the constituent scores (RT and PE) is levelled off by the aggregation of a large number of trials per condition, in accordance with the central limit theorem.

With respect to IES, the present findings are somewhat mixed. On the one hand, it is clear that effect size in IES correlated less strongly with RT effects, but equally strongly with PE effects as LISAS did. On the other hand, in the signal detection analysis IES performed at the same level as LISAS. In the simulations reported by Vandierendonck (2017), IES performed overall more poorly than both RCS and LISAS. One important difference between that study and the present one concerns the fact that absolute values of PE were smaller in the present study (0.05–0.09) than in the simulation study (0.10–0.20). As the proportion of errors increases, the denominator in Equation (1) becomes smaller, leading to larger values for IES, with as a consequence that a positive skewness is induced as the proportion of errors becomes larger (cf. Bruyer & Brysbaert, 2011) and this may result in more conservative estimates of the effects.

So far this discussion seems to suggest that usage of RCS (and throughput) is best avoided, and that for IES one should take care not to use it with error proportions above 0.10. Although such issues have not been raised so far for LISAS, the advice formulated by Bruyer and Brysbaert (2011) to always inspect the composing measures when considering the usage of an integrated speed-accuracy measure remains valid in all circumstances. Such an inspection could be guided by questions such as whether the effects of speed and accuracy are compatible and in the same direction, and whether there are any speed-accuracy trade-offs.

The present application of the integrated measures to existing data sets suggests that such analyses in addition to or in follow-up of simulation studies is useful. Note that the shortcomings noted here with respect to RCS remained under the radar of earlier simulation work (Hughes et al., 2014; Vandierendonck, 2017). Nevertheless, the present study suffers from some limitations. As a first issue, it must be pointed out that as yet not much is known about how integrated measures can be helpful when speed-accuracy trade-off comes into play. From a previous study, it is known that when SAT is unrelated to the main factor of the study, the integrated measures studied here (but not the measures based on the binning technique) keep their efficiency. Also in the data sets in the present article, SAT was not involved. Therefore, the question what happens to integrated speed-accuracy measures if effects on RT and PE are affected by SAT remains to be answered. Are integrated measures indeed of any use in situations where speed-accuracy trade-off interacts with the main manipulations, and if so what are the boundary conditions for their usage?

In recent studies of integrated measures (except for the study by Hughes et al., 2014), an experimental methodology is used. Given the differences between the experimental methodology and the methods used in studies of individual differences, the question arises whether the characteristics of the integrated measures as spelled out here and in previous studies also apply to correlational research. It cannot be simply assumed that any integrated measure that performs well in an experimental context, will also be useful in the measurement of individual differences. In the context of latent variable studies, it would seem straightforward to define a latent variable “performance” that combines speed and accuracy, but that does not provide a general solution for all types of correlation studies.

Clearly, further research regarding the influence of SAT and the utility of integrated measures in correlational research is more than welcome. As the usage of integrated measures has recently started to increase, these questions do not seem to be merely academic concerns but may result in new methodological approach to help shape the field of cognitive research.

## Conclusion

Of the integrated measures available for use, at present only LISAS and IES are sufficiently reliable and efficient for use with the provision that IES is best avoided when error proportions exceed 0.10, while RCS introduces to much variability resulting in spurious effects. In view of the variability of effects present in RT and PE, it remains recommendable to check the RT and PE effects before applying integrated measures. However, one should also bear in mind that (a) integrated measures tend to yield larger effect sizes than RT and PE, and if integrated measures are used, there is no need to separately report RT and PE analyses, which reduces the number of statistical tests by 50 percent.

## Data Accessibility Statement

The data used in the present article are accessible at https://doi.org/10.5281/zenodo.962308 for data set 1 and at https://doi.org/10.5281/zenodo.963227 for data set 2.

## Footnotes

If the number of observations per condition is too small, more stable estimates of the standard deviation can be obtained by grouping conditions, or by taking the standard deviation per subject. In the present paper, the standard deviations are calculated per condition.

Although the situation considered here raises the same problems as a speed-accuracy trade-off, it is different because opposing effects in speed and accuracy may occur apart from strategical attempts to adapt speed and accuracy. The problem of speed-accuracy trade-off will be addressed below.

Four planned contrasts were used, namely 3 orthogonal contrasts testing complete repetition v. one or more changes (loose structure), change in one v. two components (componential view), task switch v. dimension switch, and a fourth contrast for the hierarchical view contrasting a complete switch with a task switch only.

The present analyses are based on real data for which it is not known whether an effect is true or not. This may be considered as an important drawback. However, it should be noted that although the true effect is known in analyses based on simulations, the analysis of the data generated in these simulations faces the same problems, namely that in some samples the RT and/or PE effect is not reliable although it is known to be present. In order to evaluate whether an integrated measure adequately integrates the RT and PE information available in the sample, the effect sizes of the integrated measures have to be compared to those of RT and PE. In these circumstances, a relatively large and reliable effect size in an integrated measure, while there is no trace of effect in RT and PE would no doubt be considered as suspect. In other words, although the real effect is known, the analyses based on the data in the samples has to take into account whether the effect was detected by the composing measures, speed and accuracy, just like in situations where the real effect is not known.

## Author Information

The data reported in this article were collected by Evelien Christiaens and Bjørn Van Loy in the context of research projects of the author.
